# Longitudinal association between physical activity and the risk of incident metabolic syndrome in middle-aged adults in Germany

**DOI:** 10.1038/s41598-022-24052-5

**Published:** 2022-11-12

**Authors:** Laura Cleven, Janina Krell-Roesch, Steffen C. E. Schmidt, Anna Dziuba, Klaus Bös, Darko Jekauc, Alexander Woll

**Affiliations:** 1 Institute of Sports and Sports Science, Karlsruhe Institute of Technology, Engler-Bunte-Ring 15, 76131 Karlsruhe, Germany; 2 Institute of Sports Sciences, Goethe University Frankfurt, Ginnheimer Landstraße 39, 60487 Frankfurt, Germany

**Keywords:** Diseases, Risk factors, Disease prevention

## Abstract

We examined the longitudinal association between physical activity (PA) and the risk of incident metabolic syndrome (MetS) among middle-aged, community-dwelling adults, including 591 individuals (314 females; mean (SD) age, 43.8 (8.5) years) who were free of MetS at baseline. Habitual and sports-related PA was assessed by a self-reported questionnaire. MetS was defined based on HDL-cholesterols, triglycerides, glucose or HbA1c, blood pressure, and waist circumference. We calculated Cox proportional hazard ratios (HR) and 95% confidence intervals (CI) using regression analyses. Over a mean follow-up of 12.5 years, 205 participants developed incident MetS. Four different sports-related PA measures were associated with a decreased risk of incident MetS: (1) Engaging in ≥ 75 min/week (HR 0.71, 95% CI 0.53–0.94), (2) maintaining a continuously high amount from baseline to follow-up of ≥ 75 min/week (HR 0.66, 95% CI 0.46–0.94), (3) starting from < 150 min/week at baseline to ≥ 150 min/week at follow-up (HR 0.65, 95% CI 0.45–0.94), and (4) increasing from < 16.6 MET-hours/week at baseline to ≥ 16.6 MET-hours/week at follow-up (HR 0.47, 95% CI 0.31–0.71). Thus, maintaining, starting or increasing sports-related PA is associated with a lower risk of incident MetS.

## Introduction

Metabolic syndrome (MetS) refers to a cluster of cardiometabolic risk factors, including abdominal obesity, elevated blood glucose, elevated blood pressure, or dyslipidemia (elevated triglycerides and lowered high-density lipoprotein cholesterol; HDL)^1^, and is considered a non-communicable disease (NCD). NCDs have a major impact on economies and societies worldwide and have been identified as the leading reason for 73% of all global deaths, with 28.8 million deaths attributed to risk factors such as high blood pressure, high blood glucose, or high body mass index (BMI)^2–4^. The prevalence of MetS has increased over the past decades^5^. Therefore, it is critical to identify potential preventive factors and mechanisms for MetS.

In adults, decreased metabolic risk factors such as hypertension, overweight and obesity, or hyperglycemia, as well as favorable health-related behaviors such as limited tobacco or alcohol consumption, plant-based diet, and physical activity (PA) may be effective in preventing new onset of MetS. A growing body of research has reported a positive effect of PA on cardiometabolic risk factors, e.g., being physically active may reduce body weight and blood pressure, elevate high-density lipoprotein-cholesterols, lower triglycerides, and improve insulin resistance^6–10^.

The World Health Organization (WHO) recommends at least 150 min of moderate to vigorous PA, or 75 min of vigorous-intensity PA per week to achieve health benefits^11^, which is equivalent to at least 8.3–16.6 metabolic equivalent-hours (METh) per week (= 500–1000 MET-minutes per week)^12^. However, it remains unknown as to how much PA is needed to reduce the onset of MetS, or whether changes in PA behavior are related to the risk of incident MetS.

The aim of this study was thus to examine the longitudinal associations between various PA variables (i.e., habitual PA level, sport-related PA level (minutes and METh), and change in PA behavior over time) and the risk of incident MetS among middle-aged males and females in a community-based sample from South-Western Germany over a period of 29 years.

## Methods

### Study design and population

‘Gesundheit zum Mitmachen’ is an ongoing, community-based longitudinal cohort study of middle-aged and older adults living in the city of Bad Schönborn in South-Western Germany with six measurement points (1992, 1997, 2002, 2010, 2015, 2021). Detailed information about the design and methods of the study has been described elsewhere^13,14^. The study was approved by the ethics committee of the Karlsruhe Institute of Technology (KIT), Germany and was conducted in accordance with the Declaration of Helsinki. All methods were performed in accordance with the relevant guidelines and regulations.

Briefly, individuals residing in Bad Schönborn, Germany were randomly selected from the local residents’ registration offices and were invited to participate in the study. Participants who took part in the study at least once were re-invited for every measurement point, and new samples of participants aged 33–37 years were included at each measurement time point to prevent sample attrition. Participation in the study was voluntary and all participants provided written informed consent. At each of the six measurements points, participants provided information about self-rated health status, habitual and sports-related PA, and sociodemographic information through a self-reported questionnaire, underwent an objective health status examination conducted by a licensed physician, and had a fasting blood sample drawn.

**Figure 1:**
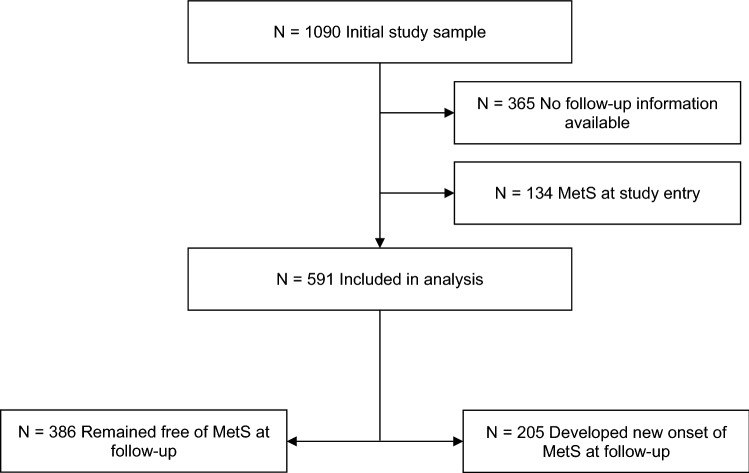
Flow chart of study participation. N = number of participants.

For the current analysis, from a total of 1090 individuals who participated in this study, we excluded participants with pre-existing MetS (i.e., outcome of interest) at baseline (N = 134) and individuals with no longitudinal data information available (N = 365). A total of 591 participants were thus included in the final analysis (please refer to Fig. 1 for a flow chart).

### Physical activity assessment (predictor variables)

#### Habitual PA

We calculated an index to quantify the amount of habitual PA at baseline (minutes per week) as indicator for recreational PA level (thus deliberately excluding sports activity participation) by creating a sum index that was derived from participants‘ self-reported information about daily minutes of walking, biking for transportation, as well as working in the household and gardening. For statistical analysis, the sample was divided into two groups (i.e., inactive: < 75 min/ week, and active: ≥ 75 min/ week) as well as three groups (i.e., low: < 75 min/ week, medium: 75–149 min/ week, and high: ≥ 150 min/ week) based on the calculated index.

#### Sports-related PA

An index for the volume of sports-related PA at baseline was calculated from information provided by participants about frequency (i.e., number of weekly exercise sessions), duration (i.e., minutes per session), intensity (i.e., not very intense, moderately intense with some sweating, and highly intense with much sweating), and type of weekly sports-related PA^15^. For statistical analyses, the sample was divided into two groups (i.e., inactive: < 75 min/ week, active: ≥ 75 min/ week) based on the index. In a next step, we also created three groups based on the global recommendation on PA level (i.e., low: < 75 min/ week, medium: 75–149 min/ week, high: ≥ 150 min/ week)^16^. Furthermore, to consider the intensity level of the activity, every type of sport was assigned a specific MET-value^17^, and by multiplication with the time spent carrying out the respective activity, sports-related PA level in MET-hours (METh) per week at baseline was calculated. For statistical analysis, in the sample was divided into two groups (i.e., not active: < 8.3 METh/ week, active: ≥ 8.3 METh/ week), as well as three groups (not active: < 8.3 METh/ week, moderately active: 8.3–16.5 METh/ week, highly active: ≥ 16.6 METh/ week) based on the sports-related PA level.

#### Change of sports-related PA categories (volume: minutes and METh)

To assess the change in minutes of sports-related PA (per week), differences in PA level between baseline and the latest follow-up examination were calculated individually for each participant. Four categories by applying two different thresholds, i.e., 75 and 150 min per week, respectively, based on WHO guidelines on physical activity and sedentary behavior^11^ were calculated. The four categories were *Stable inactive* (i.e., individuals who continuously reported less than 75/ 150 min/ week sports-related PA); *Quits activity* (i.e., participants who reported more than 75/ 150 min/ week sports-related PA at baseline but not follow-up); *Starts activity* (i.e., individuals who reported less than 75/ 150 min/ week sports-related PA at baseline but more than 75/ 150 min/ week at follow-up); and *Stable active* (i.e., participants who reported continuously more than 75/ 150 min/ week of sports-related PA). Similarly, to assess the change in METh of sports-related PA, differences in METh level at baseline examination compared to the latest follow-up examination were calculated. Then four groups were created: *Stable low* (i.e., individuals who continuously reported less than 16.6 METh/ week sports-related PA), *Decreasing* (i.e., participants who reported more than 16.6 METh/ week sports-related PA at baseline but not at follow-up); *Increasing* (i.e., individuals who reported less than 16.6 METh/ week sports-related PA at baseline but more than 16.6 METh/ week at follow-up); and *Stable high* (i.e., participants who reported continuously more than 16.6 METh/ week sports-related PA).

All information regarding habitual and sports-related PA at baseline examination and follow-up was assessed by a self-reported questionnaire (test–retest reliability after two weeks: *r* > 0.90, *α* = 0.94)^18^.

### Metabolic syndrome assessment (Outcome of interest)

At baseline and each follow-up examination, the status of MetS was assessed by self-reported medication and/or through examination by a licensed physician.

#### Metabolic syndrome

According to the Joint Interim Statement harmonized criteria, we defined having or developing new onset of MetS, when at least three out of five risk factors (i.e., elevated blood glucose, elevated blood pressure, lowered HDL, elevated triglycerides, or abdominal obesity) were present at baseline examination or at follow-up^1^.

#### Elevated blood glucose

Elevated blood glucose was determined by a physician at baseline and each follow-up examination based on blood-glucose levels (i.e., for measurement points 1992 to 2015: fasting blood-glucose level ≥ 100 mg/dL^1^ or non-fasting blood-glucose level ≥ 200 mg/dL^19^; and for measurement in 2021: HbA1c ≥ 6,5%^19^). Furthermore, a participant was classified as having elevated blood glucose, if a diabetes diagnosis had been made previously by a physician or if the participant was on diabetes medication, i.e., participants were asked: Do you take any medication to lower blood-glucose levels?

#### Elevated blood pressure

Blood pressure was assessed at baseline and each follow-up examination by standardized measurement. A participant was classified as having elevated blood pressure if the systolic value was greater than 130 mmHg or if the diastolic value was greater than 85 mmHg in the measurement. Furthermore, a participant was classified as having elevated blood pressure if hypertension diagnosis had been made previously by a physician, or if the participant was on blood pressure medication^1^, i.e., participants were asked: Do you take any medication to lower your blood pressure?

#### Blood Lipids (triglycerides and high-density lipoprotein-cholesterol)

Blood lipids were assessed at baseline and at each follow-up examination by standardized measurement. Participants were classified as having high triglycerides when the levels were greater than 150 mg/dL, and as having low HDL-cholesterol when the levels were lower than 40 mg/dL for males and lower than 50 mg/dL for females, respectively^1^. Furthermore, a participant was classified as having critical level of blood lipids (i.e., high triglycerides or low HDL-cholesterol) if the diagnosis had been made previously by a physician or if the participant was on medication for the respective condition, i.e., participants were asked: Do you take any medication to lower blood lipids?

#### Abdominal Obesity

Waist circumference (in cm) was assessed at baseline and at each follow-up examination by standardized measurement. A participant was classified as having abdominal obesity when the waist circumference was greater than 102 cm for males and greater than 88 cm for females, respectively^20,21^.

#### Assessment of confounders

Traditional demographic variables (e.g., age and sex) were assessed though self-reported questionnaire. We also determined socio-economic status (SES) based on information provided by participants about formal education and professional status of themselves or their significant others (usually the spouse), if participants were not working. Four SES categories were used for statistical analysis, i.e., low, low/medium, medium/high and high SES^14,22^.

### Statistical analysis

Selected sociodemographic, behavioral, and health-related characteristics of the participants were used to characterize the cohort at baseline by using means (M) and standard deviation (SD). Cox proportional hazards regression models were calculated to examine the associations between various PA predictor variables (i.e., volume of habitual and sports-related PA at baseline, and change in volume of sports-related PA level from baseline to follow-up; variables were nominal coded or dummy coded), and the risk of incident MetS. For the analysis, only participants who were free of MetS at baseline and had at least one follow-up measurement were included. Date of entry in the study was used as baseline and was determined individually for each participant. Follow-up time (in months) was calculated individually for each participant from baseline until the first event (i.e., incident MetS) occurred or loss to follow-up, whichever happened first. Missing values were treated as missing at random, which yielded in a pairwise deletion of cases with at least one missing value among either predictor or outcome variable for each analysis. Numbers of missing values are provided in the result table for each category of PA, respectively. We calculated Hazard Ratios (HRs) and 95% confidence intervals (CI) based on regression analyses for each category of PA as compared to the least active group which was always set as reference group. For each predictor, we ran two sets of models: unadjusted models (Model 1), and models adjusted for traditional confounding variables, i.e., age, sex and SES (Model 2). Analyses were performed using SPSS software version 27, and using the conventional alpha level of 0.05 to determine statistical significance. A HR > 1.0 was considered as indicating an elevated risk for incidence of MetS, a HR < 1.0 was considered as indicating a reduced risk for incidence of MetS.

## Results

### Sample characteristics

Baseline demographics of study participants are shown in Table 1. Data of participants who were free of MetS at baseline and with follow-up information were available on 591 individuals. Mean age at baseline was 43.8 (SD 8.5) years, and 53.1% of participants were female. Habitual PA was performed for a mean of 341.37 (SD 266.05) min/week. Sports-related PA was performed for a mean of 101.84 (SD 133.58) min/week, and with a mean of 12.04 (SD 15.96) METh/ week, and it was most frequently performed for less than 75 min/ week (53.4%). Please refer to Table 2 for an overview of frequencies with regard to PA change variables from study entry to the latest follow-up examination.

**Table 1:** Participant demographics at baseline.

	Total	Males	Females
**N**	591	277	314
**Sex (%)**	–	46.9	53.1
**Age [years], mean (SD)**	43.81 (8.53)	43.91 (8.61)	43.71 (8.46)
**SES (%)**			
low	13.2	12.0	14.2
Low/medium	20.2	19.7	20.7
Medium/high	43.4	40.1	46.3
high	23.2	28.1	18.8
**Elevated waist circumference, %**	15.8	14.2	17.5
**Elevated triglycerides, %**	7.6	11.9	3.8
**Reduced HDL, %**	12.4	10.8	13.7
**Elevated blood pressure, %**	50.8	62.1	40.8
**Elevated blood glucose, %**	1.5	2.5	0.6
**BMI [kg/m**^**2**^], **mean (SD)**	24.97 (3.41)	25.89 (2.96)	24.15 (3.57)
Normal (< 25) (%)	56.6	43.8	6.8
Overweight (25–29) (%)	35.4	47.5	24.8
Obese (≥ 30) (%)	8.0	8.7	7.3
**Habitual PA [min/week], mean (SD)**	341.37 (266.05)	368.80 (295.94)	317.27 (234.53)
Low (< 75) (%)	5.2	5.1	5.4
Medium (75–149) (%)	24.0	23.5	24.5
High (≥ 150) (%)	70.7	71.5	70.1
**Sports-related PA [min/week], mean (SD)**	101.84 (133.58)	106.95 (143.57)	97.31 (124.14)
Inactive (< 75) (%)	53.4	51.3	55.3
Medium (75–149) (%)	23.1	25.6	20.8
High (≥ 150) (%)	23.6	23.1	24.0
**Sports-related PA [METh/week], mean (SD)**	12.04 (15.96)	12.83 (17.10)	11.35 (14.87)
Low (< 8.3) (%)	54.0	50.2	57.3
Medium (8.3–16.5) (%)	21.2	24.9	17.8
High (≥ 16.6) (%)	24.9	24.9	24.8
**Follow-up time [years], mean (SD)**	12.47 (8.30)	12.95 (8.48)	12.05 (8.13)

*BMI* body mass index, *kg* kilograms, *m* meters, *METh* metabolic equivalent hours, *min* minutes, *N* number of participants, *PA* physical activity, *SD* standard deviation, *SES* socio-economic status.

Elevated waist circumference: ≥ 102 cm for males, ≥ 88 cm for females; elevated triglycerides: ≥ 150 mg/dL and/or specific medication; reduced HDL-cholesterols: < 40 mg/dL for males, < 50 mg/dL for females and/or specific medication; elevated blood pressure: systolic value ≥ 130 mmHg or diastolic value ≥ 85 mmHg and/or specific medication; elevated blood glucose: fasting blood-glucose level ≥ 100 mg/dL or non-fasting blood-glucose level ≥ 200 mg/dL or HbA1c ≥ 6,5% and/or specific medication.

**Table 2:** Frequencies with regard to PA change variables from study entry to the latest follow-up examination.

	Total	Males	Females
**Change in sports-related PA category over time [min/week], N (%) (missing N = 13)**			
Stable inactive (< 75 to < 75)	178 (30.8)	79 (29.3)	99 (32.1)
Quits activity (≥ 75 to < 75)	63 (10.9)	34 (12.6)	29 (9.4)
Starts activity (< 75 to ≥ 75)	131 (22.7)	60 (22.2)	71 (23.1)
Stable active (≥ 75 to ≥ 75)	206 (35.6)	97 (35.9)	109 (35.4)
**Change in sports-related PA over time [min/week], N (%) (missing N = 13)**			
Stable inactive (< 150 to < 150)	308 (53.3)	148 (54.8)	160 (51.9)
Quits activity (≥ 150 to < 150)	50 (8.7)	24 (8.9)	26 (8.4)
Starts activity (< 150 to ≥ 150)	134 (23.2)	61 (22.6)	73 (23.7)
Stable active (≥ 150 to ≥ 150)	86 (14.9)	37 (13.7)	49 (15.9)
**Change in sports-related PA category over time [METh/week], N (%) (missing N = 6)**			
Stable low (< 16.6 to < 16.6)	326 (55.7)	154 (56.4)	172 (55.1)
Decreasing (≥ 16.6 to < 16.6)	58 (9.9)	24 (8.8)	34 (10.9)
Increasing (< 16.6 to ≥ 16.6)	114 (19.5)	52 (19.0)	62 (19.9)
Stable high (≥ 16.6 to ≥ 16.6)	87 (14.9)	43 (15.8)	44 (14.1)

*METh* metabolic equivalent hours, *min* minutes, *N* number of participants, *PA* physical activity.

Of all participants included in the analysis, 266 (45.0%) had one follow-up assessment, 128 (21.7%) participants had two follow-up assessments, 97 (16.4%) participants had three follow-up assessments, 61 (5.6%) had four follow-up assessments, and 39 (3.6%) participants had 5 follow-up assessments. The mean (SD) time of follow-up assessments was of 2.1 (1.3). After a mean follow-up time of 12.5 (SD 8.3, range 5–29) years, 205 (34.7%) participants developed incident MetS and 386 (65.3%) remained free of MetS at follow-up (please refer to Fig. 1) with 7373 person-years (sum of the time spans for each participant under observation). The risk of incident MetS did not statistically significantly differ between males (51.2%) and females (48.8%). The mean age at which MetS was first diagnosed was 56.8 (SD 9.5; range: 38–81) years. Cases of incident MetS were more frequent in older than younger persons (75.1% cases of MetS occurred in participants aged > 50 years). Participants across all SES developed incident MetS, with most incident MetS cases (N = 73, 35.6%) in the medium to high SES group. 134 participants with a mean age of 49.7 (SD 10.6; range: 33–77) years were not included in this analysis as they had prevalent MetS at study entry.

### Association between physical activity (habitual and sports-related) at baseline and incident metabolic syndrome

After adjusting for age, sex and SES, engaging in more than 75 min of sports-related PA per week at baseline was associated with a 29% reduced risk of incident MetS (HR 0.71, 95% CI 0.53–0.94, p = 0.02), compared to participants who reported engaging in less than 75 min of sports-related PA per week (please refer to Table 3). Whereas, participating in sports-related PA between 75–149 min/ week at baseline was associated with a 38% decreased risk of incident MetS (HR 0.62, 95% CI 0.43–0.91, p = 0.01) compared to sports-related PA carried out for less than 75 min/ week. For high sports-related PA level (≥ 150 min/ week), there was a trend for a reduced risk of incident MetS (HR 0.80, 95% CI 0.56–1.13, p = 0.20), albeit not statistically significant.

**Table 3:** Association between PA variables and the risk of incident MetS.

No. with incident metabolic syndrome (N = 205, 34.7%)						
	No. at risk (N = 591)	No. incident MetS (% of No. at risk)	Model 1 unadjusted hazard ratio (95% CI)	*p*-value	Model 2 adjusted hazard ratio (95% CI)	*p*-value
**Amount of habitual PA level at baseline [min/week]**						
Inactive (< 75)	31	11 (1.9)	ref		ref	
Active (≥ 75)	560	194 (32.8)	1.05 (0.57–1.92)	0.88	1.15 (0.61–2.18)	0.66
**Amount of habitual PA level at baseline [min/week]**						
Low (< 75)	31	11 (1.9)	ref		ref	
Medium (75–149)	142	40 (6.8)	0.80 (0.41–1.57)	0.52	1.00 (0.50–2.01)	0.99
High (≥ 150)	418	154 (26.1)	1.14 (0.62–2.10)	0.68	1.20 (0.63–2.27)	0.58
**Volume of sports-related PA level at baseline [min/week] (missing N = 1)**						
Inactive (< 75)	315	126 (21.3)	ref		ref	
Active (≥ 75)	275	79 (13.4)	**0.62 (0.47–0.83)**	** < 0.01**	**0.71 (0.53–0.94)**	**0.02**
**Volume of sports-related PA level at baseline [min/week] (missing N = 1)**						
Low (< 75)	315	126 (21.3)	ref		ref	
Medium (75–149)	136	36 (6.1)	**0.53 (0.37–0.77)**	** < 0.01**	**0.62 (0.43–0.91)**	**0.01**
High (≥ 150)	139	43 (7.3)	0.73 (0.52–1.04)	0.08	0.80 (0.56–1.13)	0.20
**Change in sports-related PA category over time [min/week] (missing N = 13)**						
Stable inactive (< 75 to < 75)	178	73 (12.4)	ref		ref	
Quits activity (≥ 75 to < 75)	63	18 (3.0)	**0.59 (0.35–0.99)**	**0.05**	0.71 (0.42–1.20)	0.20
Starts activity (< 75 to ≥ 75)	131	51 (8.6)	0.75 (0.52–1.07)	0.12	0.84 (0.59–1.21)	0.35
Stable active (≥ 75 to ≥ 75)	206	61 (10.3)	**0.55 (0.39–0.76)**	** < 0.01**	**0.66 (0.46–0.94)**	**0.02**
**Change in sports-related PA category over time [min/week] (missing N = 13)**						
Stable inactive (< 150 to < 150)	308	119 (20.1)	ref		ref	
Quits activity (≥ 150 to < 150)	50	18 (3.0)	0.88 (0.54–1.44)	0.61	0.94 (0.57–1.54)	0.79
Starts activity (< 150 to ≥ 150)	134	40 (6.8)	**0.56 (0.39–0.80)**	** < 0.01**	**0.65 (0.45–0.94)**	**0.02**
Stable active (≥ 150 to ≥ 150)	86	26 (4.4)	0.70 (0.46–1.07)	0.10	0.77 (0.50–1.18)	0.23
**Volume of sports-related PA at baseline [METh/week]**						
not active (< 8.3)	319	123 (20.8)	ref		ref	
active (≥ 8.3)	272	82 (13.9)	**0.74 (0.56–0.98)**	**0.04**	0.80 (0.60–1.06)	0.13
**Volume of sports-related PA at baseline [METh/week]**						
not active (< 8.3)	319	123 (20.8)	ref		ref	
moderately active (8.3–16.5)	125	36 (6.1)	**0.67 (0.46–0.98)**	**0.04**	0.75 (0.52–1.10)	0.14
highly active (≥ 16.6)	147	46 (7.8)	0.81 (0.57–1.13)	0.21	0.84 (0.60–1.18)	0.32
**Change in sports-related PA category over time [METh/week] (missing N = 6)**						
Stable low (< 16.6 to < 16.6)	326	131 (22.2)	ref		ref	
Decreasing (≥ 16.6 to < 16.6)	58	19 (3.2)	0.79 (0.49–1.29)	0.35	0.82 (0.51–1.33)	0.43
Increasing (< 16.6 to ≥ 16.6)	114	27 (5.6)	**0.43 (0.28–0.64)**	** < 0.01**	**0.47 (0.31–0.71)**	** < 0.01**
Stable high (≥ 16.6 to ≥ 16.6)	87	27 (5.6)	0.70 (0.46–1.06)	0.09	0.73 (0.48–1.11)	0.14

*CI* confidence interval, *METh* metabolic equivalent hours, *min* minutes, *N* number of participants, *No* Number, *PA* physical activity, *ref* reference group, *SES* socio-economic status. Model 1: unadjusted; Model 2: adjusted for age, sex und SES.

Significant values are in [bold]

With regard to METh of sports-related PA, being physically active with more than 8.3 METh/ week at baseline was associated with a reduced risk of incident MetS (HR 0.74, 95% CI 0.56–0.98, p = 0.04) only in the unadjusted model, and the association was no longer statistically significant after adjusting for confounding variables (Model 2: HR 0.80, 95% CI 0.60–1.06, p = 0.13).

Habitual PA was not statistically significantly associated with the risk of incident MetS in our dataset.

### Association between change of sports-related physical activity (minutes and METh) categories over time and incident metabolic syndrome

After adjusting for age, sex and SES, being stable active (stable ≥ 75 min/ week from baseline to follow-up) was associated with a 34% reduced risk of incident MetS (HR 0.66, 95% CI 0.46–0.94, p = 0.02) compared to being stable inactive (please refer to Table 3). Similarly, for participants who were stable active (stable ≥ 150 min/ week from baseline to follow-up), a trend for a reduced risk of incident MetS was observed (HR 0.77, 95% CI 0.50–1.18, p = 0.23), albeit not statistically significant. Furthermore, participants who reported starting to engage in sports-related PA from < 150 min/ week at baseline to ≥ 150 min/ week at follow-up, had a 35% reduced risk of incident MetS (HR 0.65, 95% CI 0.45–0.94, p = 0.02) as compared to participants who continuously remained inactive. In addition, increasing sports-related PA from < 16.6 METh per week at baseline to ≥ 16.6 METh per week at follow-up, was associated with a 53% reduced risk of incident MetS (HR 0.47, 95% CI 0.31–0.71, p < 0.01) compared to participants with a stable low sports-related PA. For participants who reported a stable high sports-related PA (stable ≥ 16.6 METh/ week), a trend for a reduced risk of incident MetS was observed (HR 0.73, 95% CI 0.48–1.11, p = 0.14), albeit not statistically significant.

## Discussion

Our study adds to the current body of evidence on an association between higher PA at baseline and a decreased risk of incident MetS in middle-aged adults, with risk reductions ranging from 29 to 38% for sports-related but not habitual PA. In addition, even though the association between the volume of sports-related PA carried out for ≥ 150 min per week and incident MetS was not statistically significant, the point estimate was below 1.0 indicating a trend for a reduced risk of incident MetS. The WHO currently recommends engaging in at least 75 to 150 min per week of PA for adults. Based on our data, even a threshold of engaging in sports-related PA for at least 75 min per week may be associated with a reduced risk of incident MetS. Researchers and stakeholders may thus want to consider this threshold in the design and conduct of PA programs or public health strategies aimed at promoting metabolic health in the general population.

In addition, favorable changes in sports-related PA from baseline to follow-up were associated with reduced risks of incident MetS. Our results show that engaging continuously in sports-related PA for ≥ 75 min/ week from baseline to follow-up was associated with a 34% reduced risk of incident MetS as compared to being permanently inactive from baseline to follow-up. The point estimates for the association between engaging continuously in ≥ 150 min/ week of sports-related PA and incident MetS were below 1.0 which is indicative of a reduced risk for new onset of MetS, albeit not statistically significant. Thus, these findings underline the importance of maintaining continuous engagement in sports-related PA across the lifespan in order to achieve desirable health benefits with regard to MetS risk reduction. Nevertheless, our results also show that starting engaging in sports-related PA from < 150 to ≥ 150 min/ week was associated with a 35% reduced risk of incident MetS compared to participants who continuously remained physically inactive. This indicates that it may never be too late to start engaging in sports-related PA, albeit higher volumes seem to be necessary when starting at older age and/ or later date.

Furthermore, aside from the volume of sports participation in minutes per week as described above, METh also appear to be important. For example, we observed that increasing PA intensity level from < 16.6 METh/ week at baseline to ≥ 16.6 METh/ week at follow-up was statistically significantly associated with a 53%-reduced risk of incident MetS.

Overall, our results are in line with previous longitudinal studies. For example, the Tromsø Study with a total sample of 17,014 individuals, reported a significant inverse association between leisure-time PA at baseline and incident MetS, with a more than 40% reduced risk of incident MetS over a mean follow-up of 13.8 years (with a maximum of 22 years)^23^. Whereas, investigators from the Atherosclerosis Risk in Communities study reported only a weak association between PA and incident MetS in a sample of 9,359 males and females and based on a follow up time of 6 years^24^. Furthermore, two meta-analyses showed that high as compared to low PA levels were associated with a reduced risk of MetS, and risk reductions ranged from 10–20%^25,26^. Conflicting results have been reported in the literature with regard to associations between PA METh at baseline and risk of incident MetS, and the association may also be sex-specific. For example, a trend for a decreased risk of MetS with increasing dose of PA was reported in men, whereas a decreased risk in women was observed only for high or very high levels of baseline PA in a sub-cohort of the Japan Epidemiology Collaboration on Occupational Health Study with a total of 22,383 participants included and a mean follow-up time of 4.1 years (maximum 5 years)^27^. Furthermore, the Copenhagen City Heart Study including 3,968 men and women, revealed that moderate and high but not light PA volumes were associated with a lower risk of MetS, with about 50% risk reduction over a follow-up time of 10 years^28^. With regard to the association between changes in PA levels from study entry to follow-up and incident MetS, results of a longitudinal study conducted in Brazil showed a 65% reduced risk to develop MetS for permanently active participants compared to permanently inactive participants, whereas starting PA reduced the risk of incident MetS about 45%^29^. Similarly, the Coronary Artery Risk Development in Young Adults study, including 4,192 men and women, reported that maintaining regular PA over time was associated with a 35% reduced risk of MetS during a mean follow-up time of 13.6 years (with a maximum up to 15 years)^30^.

A major strength of our study is the longitudinal design with a long follow-up period of 29 years. To the best of our knowledge, only a few studies exist with such a long follow-up time (e.g., Harvard Alumni Study^31^, Framingham Heart Study^32^, National Health and Nutritional Examination Survey^33^, Nurses’ Health Study^34^). In addition to previous studies, our analysis focused not only on the volume of sports-related PA at baseline, but also considered habitual activity and change in sports-related PA during follow-up. Furthermore, self-reported health status of participants was augmented by a health examination performed by a licensed physician.

Limitations of our study pertain to the rather small sample with participants predominantly having medium to high SES. Thus, our results may not be transferable to communities of middle-aged adults with lower SES. In addition, PA was assessed by a self-reported questionnaire which may be prone to recall bias, i.e., PA may have been over- or underestimated by participants. However, the questionnaire used in our study has been tested for reliability (test-reliability for two weeks *r* > 0.90) and internal consistency (α = 0.94)^18^. Furthermore, we did not examine changes in PA between childhood or youth and adulthood, or between early and middle adulthood. It is known that participation in PA varies across the lifespan, e.g., sports behavior is rather stable from childhood to youth^35^, whereas PA behavior in adulthood is instable^36^. Even though our study is in line with previous studies among adults that also provided evidence of an association between higher PA levels at baseline and favorable health outcomes at later life (e.g.^37^), future research may want to focus more closely on PA behavior changes across the lifespan.

In our study, we considered new onset of MetS as the outcome of interest. However, due to its definition, the status of MetS may fluctuate over time in participants, particularly as we had several measurement points. However, the majority of participants had only one (45% of participants) or two follow-up examinations (21.7% of participants). Furthermore, future studies should thus not only focus on the first event of incident MetS in a dataset but also consider change in status of MetS. In addition, in this analysis, we only examined the potential impact of PA on MetS. However, MetS is multifaceted and incidence of MetS, as well as PA pattern, may be influenced by other behavioral and/or lifestyle-related factors such as nutrition^8,38–41^.

Furthermore, future studies should not only focus on incident MetS as the outcome of interest, but also examine the association between PA with individual factors of MetS (i.e., waist circumference, glucose, HDL, triglycerides, blood pressure). Such approach would likely yield more detailed information on the potential impact of PA on various risk factors contributing to MetS. Furthermore, even though we conducted a longitudinal study to examine the association between PA (which was considered the predictor) and incident MetS (which was considered the outcome of interest), we cannot answer the question of cause and effect, and inverse causality may thus be possible. This means that participants who are in the early stages of MetS or have metabolic risk factors not yet severe enough to be classified as MetS, may be less likely to engage in sports-related PA at baseline or may be more likely to remain inactive during follow-up, and this may explain our observed associations between higher sports-related PA at baseline or continuous engagement in sports-related PA from baseline to follow-up, with a decreased risk of incident MetS. In addition, particularly in participants with loss to follow-up due to illness or death, or those with rather short follow-up period, the likelihood of observing changes in PA over time was low; which may have led to potential bias and reduced power for our statistical analyses. Similarly, the range of PA change differs between participants depending on the individual duration of follow-up which should also be taken into account when interpreting our data. Finally, occurrence of the event (i.e., incident MetS) may not have occurred during the data collection phase of this study, and participants may develop incident MetS after the observation period. Therefore, more research is needed to untangle the longitudinal associations between PA and incident MetS, and our study also needs to be confirmed by prospective studies conducted in other communities.

In conclusion, we here provide additional evidence of an association between sports-related PA and a decreased risk of incident MetS, i.e., being physically active at baseline with an amount of at least 75 min per week, as well as remaining physically active or even starting sports-related PA during follow-up were associated with a reduced risk of incident MetS. Our data shows that PA should be carried out at a rather high volume in order to achieve favorable health outcomes, and that habitual PA was not related to the risk of incident MetS. Our findings have implications for PA promotion in middle-aged adults aimed at increasing metabolic health, and underline the importance of structured PA health promotion programs and public health strategies, even at community-based levels.

## Data Availability

The datasets generated and analyzed for the current study are not publicly available due to the strict ethical standards as required by the ethics committee of the Karlsruhe Institute of Technology, Germany. However, data may be available from the corresponding author on reasonable request.
